# “I can’t do it”: A qualitative study exploring case and contact experiences with COVID-19 contact tracing

**DOI:** 10.1186/s12889-022-14265-8

**Published:** 2022-10-25

**Authors:** Tyler Shelby, Cailin Arechiga, Amanda J. Gupta, Rachel Hennein, Christopher Schenck, Brian Weeks, Maritza Bond, Linda Niccolai, J. Lucian Davis, Lauretta E. Grau

**Affiliations:** 1 Department of Epidemiology of Microbial Diseases, Yale School of Public Health, New Haven, Connecticut, United States of America; 2 Yale School of Medicine, New Haven, Connecticut, United States of America; 3 New Haven Health Department, New Haven, Connecticut, United States of America; 4 Pulmonary, Critical Care, and Sleep Medicine Section, Yale School of Medicine, New Haven, Connecticut, United States of America; 5 Center for Methods in Implementation and Prevention Science, Yale School of Public Health, New Haven, Connecticut, United States of America; 6 Present Address: Norwalk Health Department, Norwalk, CT United States of America

## Abstract

**Background:**

Low engagement in contact tracing for COVID-19 dramatically reduces its impact, but little is known about how experiences, environments and characteristics of cases and contacts influence engagement.

**Methods:**

We recruited a convenience sample of COVID-19 cases and contacts from the New Haven Health Department’s contact tracing program for interviews about their contact tracing experiences. We analyzed transcripts thematically, organized themes using the Capability, Opportunity, Motivation, Behavior (COM-B) model, and identified candidate interventions using the linked Behavior Change Wheel Framework.

**Results:**

We interviewed 21 cases and 12 contacts. Many felt physically or psychologically incapable of contact tracing participation due to symptoms or uncertainty about protocols. Environmental factors and social contacts also influenced engagement. Finally, physical symptoms, emotions and low trust in and expectations of public health authorities influenced motivation to participate.

**Conclusion:**

To improve contact tracing uptake, programs should respond to clients’ physical and emotional needs; increase clarity of public communications; address structural and social factors that shape behaviors and opportunities; and establish and maintain trust. We identify multiple potential interventions that may help achieve these goals.

**Supplementary Information:**

The online version contains supplementary material available at 10.1186/s12889-022-14265-8.

## Background

Contact tracing, a non-pharmaceutical intervention used to limit transmission of a variety of infectious diseases [1, 2], was widely adopted in response to the COVID-19 pandemic [3, 4], with demonstrated reductions in case incidence [5, 6] and mortality [7] and several additional benefits including delivery of education and linkage to social support resources. However, traditional contact tracing is challenging to implement because it depends on a chain of four independent behaviors expected of cases and contacts: (1) testing, (2) answering phone calls, (3) participating in interviews with contact tracers, and (4) isolating or quarantining when indicated. Cases and contacts may engage in some, all, or none of these, yet the overall impact of contact tracing relies on their cumulative completion rate [8]. A recent evaluation of 14 U.S. COVID-19 contact tracing programs [9] found rates of interview completion among cases below 60%, a threshold defined early in the pandemic as a minimum for mitigating epidemic growth [10]. Additional studies outside the U.S. found rates of adherence to isolation and quarantine as low as 25% [11, 12].

Given the importance of contact tracing in the ongoing pandemic response, it is critical to identify and understand elements that influence participation. What is currently known about engagement in contact tracing is derived from studies of other infectious diseases, digital contact tracing, or single steps of contact tracing such as self-isolation [13–16]. Yet, many behavior change theories, models and frameworks exist to help classify elements influencing engagement in COVID-19 contact tracing and identify behavioral interventions to address them. The Capability, Opportunity, Motivation, Behavior (COM-B) model [17] is particularly suited for this objective given its linkage to the Behavior Change Wheel [17] implementation framework, and has been used to identify and select behavior-modifying interventions in other contexts [18, 19]. COM-B proposes three primary determinants (domains) of behavior: (1) *Capability*, (2) *Opportunity*, and (3) *Motivation*. These domains include sub-domains that further categorize these influences. *Capability* includes *Physical Capability* (physical strengths or abilities) and *Psychological Capability* (prerequisite knowledge, mental skills/stamina); *Opportunity* includes *Physical Opportunity* (physical environment and resources) and *Social Opportunity* (social factors, norms, relationships); and *Motivation* includes *Reflective Motivation* (intentional thought processes) and *Automatic Motivation* (impulses or emotions).

The goal of this study is to elicit, from qualitative interviews with COVID-19 cases and contacts, elements that influenced their engagement and apply an implementation mapping [20] approach using the COM-B model and Behavior Change Wheel framework to identify potential strategies to promote engagement in contact tracing.

## Methods

### Study setting

This study contributed to a multiple methods evaluation of the New Haven Health Department’s (NHHD) COVID-19 contact tracing program [21–23], which operated from March to June 2020. This program was staffed by NHHD employees, but primarily utilized university volunteers, including members of the study team (TS, CS). Volunteers signed confidentiality agreements with the NHHD allowing them to assist with contact tracing and access limited client data necessary for their assigned tasks. Multilingual volunteers were utilized to reach non-English speaking clients. New Haven, Connecticut is part of the New York Metropolitan Area and home to roughly 130,000 racially and ethnically diverse residents (30% White, 33% Black/African-American, 31% Hispanic/Latinx, and 5% Asian) [24]. Between April and June 2020, the contact tracing program reached nearly 1,300 COVID-19 cases and almost 1,100 contacts.

### Study population eligibility and recruitment

We invited consecutive adult COVID-19 cases and their close contacts if they were documented as adult clients in the NHHD contact tracing registry and successfully reached for a tracing interview within the preceding 7–28 days. We sought to interview both cases and contacts because successful contact tracing depends on the attitudes, motivations, and actions of cases as well as their contacts. We set a target recruitment of 15 cases and 15 contacts based on estimates of the number of interviews needed to reach thematic saturation [25]. Participants received a $20 gift card upon completing an interview. Enrollment continued until the NHHD’s contact tracing program ended in June 2020.

### Data collection procedures and analysis

We obtained basic demographic data (age, sex, language preference, and race/ethnicity in cases only) from the NHHD registry. Our semi-structured interview guide explored two topics: (1) experiences related to four key contact tracing behaviors (testing, answering phone calls, participating in contact tracing interviews, and adhering to isolation or quarantine) (Supplementary Fig. 1), and (2) recommendations to improve contact tracing procedures. The current analysis primarily focuses on the first topic.

The interview team included a male MD/PhD student (TS) and a female, Spanish-speaking MPH student (CA) who were trained in qualitative interviewing. They telephoned clients up to three times over a one-week period to invite them to participate in the study and left voice messages with callback numbers if clients did not answer. If invited clients did not call back within one week from the initial invitation, no additional contact was attempted. Interviews were conducted via telephone, audio-recorded, and subsequently transcribed verbatim (and translated to English, if applicable) using an automated service (Trint, London, United Kingdom). Transcripts were proofread against the recordings and corrected as needed. Participants did not review the data or study findings. TS and LG iteratively assessed the content of interviews on a weekly basis for saturation until no new themes emerged.

The coding team (TS and LG) adapted a codebook from a prior qualitative evaluation of contact tracing [23] and added new codes as needed. They independently coded all transcripts and met to resolve any coding discrepancies. They then entered the coded transcripts into ATLAS.ti (Version 8, Berlin, Germany) for analysis.

TS, JLD, and LG analyzed the coded data [26] to identify preliminary themes, and subsequently narrowed the analytic scope to the four client behaviors of interest. They classified themes and supporting quotes as barriers to or facilitators of participation in contact tracing, and organized them within all relevant COM-B domains [17].

## Results

### Study sample characteristics

Between May 25 and July 9, 2020, we telephoned 64 cases and 83 contacts of whom 35 cases and 38 contacts answered or called back, and 21 cases and 12 contacts agreed to participate. Three contacts had tested positive for COVID-19 by the time of the study interview. Participants’ median age was 41, 61% were female, and the largest racial/ethnic group in our sample was Hispanic/Latinx (48%) (Table 1), which is roughly representative of the NHHD’s client population during this timeframe [22].

**Table 1:** Participant characteristics

Characteristics (n = 33)	n (%)^a^
**Participant Type**	
Case	21 (64)
Contact	9 (27)
Contact who subsequently tested positive	3 (9)
**Age, median years (Q1, Q3)** ^***b***^	40 (32, 52)
**Gender**	
Female	20 (61)
Male	13 (39)
**Race/Ethnicity**	
Non-Hispanic White	5 (15)
Black/African American	4 (12)
Hispanic/Latinx	16 (48)
Asian	2 (6)
Native American	1 (3)
Unknown	5 (15)
**Language Spoken**	
English	21 (64)
Spanish	12 (36)

Legend

^*a*^ *Unless otherwise specified*

^*b*^ *Q1, quartile 1; Q3, quartile 3*

### Themes, facilitators and barriers

We identified seven themes that cut across the four behaviors and three COM-B domains (Table 2). While the themes were broadly similar across case and contact groups, we note relevant differences below when applicable, summarize the individual themes within each COM-B domain, and present supporting quotes in Table 3.

**Table 2:** Facilitators and barriers mapped onto behaviors, themes, and COM-B domains (Capability, Opportunity, Motivation)

Themes		Testing	Answering	Participating in Interview	Isolation/Quarantine
**Capability**					
Symptom Severity	B	-	Symptoms limit ability to answer	Symptoms limit ability to speak	Symptoms increase difficulty
	F	-	-	-	-
Essential Knowledge	B	Lacking awareness of where/when to get tested limits uptake	Cases/contacts are surprised by call due to being unaware of tracing	Lacking understanding of tracing limits participation	Lacking understanding of I/Q protocols increases confusion
	F	-	-	Education increases participation	-
**Opportunity**					
Structural Context	B	Lacking insurance or transportation impedes care seeking and testing	Language barriers limit receptiveness	Language barriers impede communication; Work/home responsibilities limit availability	Lacking food or secure/spacious housing and need for work limit feasibility
	F	In-home testing and policies increase uptake	-	Having staff who are able to speak the patient’s preferred language increases receptiveness	Organizational support, paid work leave, and spacious housing increase feasibility
Interpersonal Ties	B	-	When cases withhold contact info for any reason, they close off the possibility of outreach workers screening their contacts	-	Caregiving responsibilities make complete adherence not feasible
	F	Prompting by family/peers increases uptake	Cases alert contacts to incoming calls; Family assistance of ill cases increases feasibility	Family assistance of ill cases increases feasibility; Shared experiences reduce fears	Peer/family encouragement increases adherence; Providing food, housing, financial support increases feasibility.
**Motivation**					
Symptom Severity	B	-	-	-	Lack of symptoms reduces motivation
	F	Symptoms increase motivation	-	-	Symptoms increase motivation
Anticipated Outcomes	B	Belief that testing will not lead to support, assumed infection status, and desire to exit quarantine quickly limit uptake	Belief that answering will not lead to support limits uptake	-	-
	F	Curiosity and desire to ensure medical care increases uptake	Desire for information increases uptake	Desire for information, for medical/resource support, and to protect community increase uptake	Desire to protect community increases uptake
Trust in Authority	B	-	-	Potential for data misuse and disorganized outreach lead to fear and loss of credibility	-
	F	Trust in guidance increases uptake	Use of Caller ID limits concerns about scam callers	Caller’s advance knowledge of client birth date increases trust	Trust in guidance increases adherence
Emotional Responses	B	-	-	Shock/anxiety/anticipated stigma impede interviews; Disorganized outreach upsets clients	Boredom and loneliness negatively impact mental health
	F	-	-	Contributing to public health is gratifying; Communication skills address negative emotions	Coping strategies improve mental health; Follow-up calls provide reassurance during I/Q

**Abbreviations**:

COM-B: Capability, Opportunity, Motivation, Behavior

B: Barrier

F: Facilitator

I/Q: Isolation and quarantine

**Table 3:** Supporting quotes within each theme and COM-B domain

COM-B Domain	Theme	Quote	Behavior
**Capability**	Symptom Severity	*I had a lot of cough and I couldn’t speak…my wife just put [the tracer] on speaker and [I was] listening to [them] and she was answering for me.* (Participant 10, Case)	P
		*I [isolated] almost three weeks ‘cause I was weak and my taste buds hadn’t quite got back yet…. I lost eight pounds and I was already thin… That was the hardest part.* (Participant 2, Case)	I/Q
	Essential Knowledge	*I was surprised [to receive the call]. I didn’t expect that call at all…I didn’t know [contact tracing] was a thing.* (Participant 4, Contact)	A
		*At first, I didn’t want to give the names [of my contacts], but then when they explained to me the reason why [it] was important to them… I answered the questions.* (Participant 3, Contact who tested positive)	P
		*[I asked the tracers,] do I need two negative tests to stop self-isolation? And the [city’s tracers] said, yes, you should get retested and [the university] said they were not recommending retests.* (Participant 12, Case)	T, I/Q
**Opportunity**	Structural Context	*I answered [the contact tracing call] and I said [I was busy and] that they could call back in an hour. They never did.* (Participant 24, Contact)	P
		*[My employers] are not supporting me or paying me either. Because I’m not working. They pay you when you work.* (Participant 28, Case)	I/Q
		*The last week before I was better, I had to put a mask on and run to the closest store…because we had been running out of food.* (Participant 16, Contact who tested COVID-positive)	I/Q
		*English for me is the second language. Sometimes you have words or little bits [that are difficult] to understand exactly what the person is talking about.* (Participant 15, Contact)	P
		*It was hard to find a doctor… My family is not registered in any clinic.* (Participant 25, Contact who tested positive)	I/Q
	Interpersonal Ties	*I started having symptoms again and my wife is also a nurse in my country…she said it was necessary to do the COVID test.* (Participant 10, Case)	T
		*I told [my contacts about my positive test] …and then I told them they would probably be receiving a call [from the health department].* (Participant 12, Case)	A
		*I didn’t exactly give names and phone numbers, but I just said that it was my family… I [also] didn’t completely isolate myself because I have my kids. I was being very careful, right?* (Participant 7, Case)	A, P, I/Q
**Motivation**	Symptom Severity	*I went to the hospital the day after [symptom onset] to get the test…* (Participant 9, Case)	T
	Anticipated Outcomes	*The reason I requested a test was because I wanted to make sure I would get adequate health care. I have ulcerative colitis.* (Participant 13, Case)	T
		*What [my family] did was they went by my tests and figured they had the same thing ‘cause we were all together that Sunday [before I was diagnosed].* (Participant 2, Case)	T
		*My mom wasn’t happy with [the contact tracing calls which she didn’t answer]…she felt that a phone call wasn’t going to help her. She needed an actual doctor.* (Participant 25, Contact who tested positive)	A
	Trust in Authority	*[My wife, a case,] was a little intimidated because, although it was explained how the information would be used, a potential fear she had was the information being manipulated somehow in terms of her personal life.* (Participant 4, Contact)	P
		*When the phone rang, the number of the person and the name of the [health department] come on my tv screen. So I knew it wasn’t a scam….after [the tracer] hung up, I knew exactly what I had to do. I called my doctor and I told him. [He] put me on course, set me up with an appointment to get tested again.* (Participant 17, Contact)	A, P, T
	Emotional Responses	*I have anxiety. I got overwhelmed. And I was like, “I can’t do it [the interview].” …I [first] felt the support, but then it became annoying because they [were] calling me almost every day.* (Participant 19, Case)	P
		*It was almost like getting a phone call telling me I had AIDS… So everything for me is going to be like, you can’t do this, you can’t do that… So it was almost to the point where I could have cried when they told me because it was how people [were reacting] to it.* (Participant 2, Case)	P
		*You get up in the morning and you look around, you go back and you wash. You try to make yourself a little something to eat. You open up the door and look out. You don’t go out the door. You just look out the door. You close the door and you walk around your apartment again and you’re saying, “what in the heck am I going to do today?” …On my second week of [quarantine], I said I know I have a backyard. I have some seeds. I’m gonna make myself a little small garden in my backyard.* (Participant 17, Contact)	I/Q

**Abbreviations**

T: Testing

A: Answering phone calls

P: Participating in interviews

I/Q: Isolation and quarantine

### Capability domain

We identified two themes, *Symptom Severity* and *Essential Knowledge*, related to participants’ capacity to participate in contact tracing. *Symptom Severity* describes how COVID-19 symptoms influenced their *Physical Capability*. *Essential Knowledge* describes how knowledge about the purpose of and procedures for testing and tracing influenced their *Psychological Capability*.

#### Symptom severity theme

Several participants described how symptoms, such as shortness of breath, made it difficult or infeasible to answer phone calls or speak to contact tracers. One case was hospitalized at the time of the contact tracing call, and his daughter spoke on his behalf. Other participants noted that moderate or severe symptoms also made isolation especially difficult.

#### Essential knowledge theme

Limited awareness of COVID-19 symptoms, testing locations, or contact tracing procedures acted as a barrier to contact tracing engagement for several participants. For example, not knowing how personal data would be used or protected caused some participants, particularly cases, to be wary of fully engaging with the interview, although some contact tracers successfully addressed these concerns. Other participants were confused by quarantine and isolation instructions.

### Opportunity domain

We identified two themes, *Structural Context* and *Interpersonal Ties*, related to participants’ possibility of participating in contact tracing. *Structural Context* describes how structural factors (fixed economic, social, and policy factors) influenced *Physical* and *Social Opportunity* to participate in contact tracing. *Interpersonal Ties* describes ways in which social roles and connections with family, friends, or colleagues further influenced their *Social Opportunity*.

#### Structural context theme

Participants identified multiple structural factors including lacking transportation or receiving tracing calls at inconvenient times that hindered or delayed participant engagement. Although the NHHD had a referral system to address food, housing and other client needs, study participants frequently cited concerns about loss of income, housing instability, and food insecurity as barriers to isolation/quarantine. Isolation and quarantine were even more difficult in homes with inadequate space to allow household members to effectively separate from one another. Several participants received food from clinics and volunteer organizations, and some had access to paid leave from their workplace. One systems-level facilitator identified by several participants was policy-mandated testing, requiring testing in order to enter health care clinics or travel internationally.

*Social Opportunity* for engagement in contact tracing was influenced by access to medical providers and language services. Those lacking health insurance or established relationships with care providers experienced difficulties accessing care during isolation/quarantine. For participants whose preferred language was not English, language barriers made answering calls and participating in contact tracing interviews infeasible or challenging, although some noted that multilingual outreach workers or translation services enabled successful interaction with the program.

#### Interpersonal ties theme

Participants often described how relationships with family or friends could encourage testing and tracing behaviors and reassure participants about the contact tracing experience. Peers frequently encouraged engagement in testing or tracing, with some cases even alerting their contacts to expect tracing calls. As previously noted, family members often helped by answering phone calls for symptomatic cases and caring for those in isolation or quarantine. Interpersonal ties also hindered contact tracing efforts. Some cases did not provide tracers with information about their contacts (names and phone numbers), thereby preventing the health department from reaching them. Caregiving responsibilities at home (e.g., for children) posed additional barriers to adhering to isolation/quarantine guidelines.

#### Motivation domain

We identified some aspects of *Symptom Severity* and three additional themes, *Anticipated Outcomes*, *Trust in Authority*, and *Emotional Responses*, that related to participants’ motivation to participate in contact tracing. *Symptom Severity* describes ways in which symptoms, or lack thereof, influenced their *Reflective Motivation*. *Anticipated Outcomes* describes ways in which their beliefs in the consequences of participation, whether positive, negative, or neutral, also influenced *Reflective Motivation. Trust in Authority* is the last theme associated with *Reflective Motivation*, and it describes the influence of participants’ trust in providers and health systems. *Emotional Responses* describes ways in which participants’ emotions influenced their *Automatic Motivation*.

#### Symptom severity theme

Participants frequently described how symptoms prompted testing or isolation. By contrast, one contact without any symptoms described the quarantine experience as feeling “so abstract” because the lack of symptoms made it “hard to keep telling myself this is real.”

#### Anticipated outcomes themes

Participants varied in their expectations about the consequences of participation with contact tracing. Several tested or answered phone calls to ensure that they received adequate social or medical support, even when asymptomatic. One participant tested out of curiosity, while others assumed their status was positive based on known exposures and chose not to test. Some participants reported participating in testing and tracing mainly to prevent transmission to others. By contrast, skepticism about the benefits of testing, or fear of unwanted consequences (e.g., mandatory isolation) reduced engagement.

#### Trust in authority theme

Trust in the health system and guidelines motivated many to participate in contact tracing, while fears about misuse of data or mishandling of medical care acted as barriers. Signs of disorganization in outreach efforts, such as duplicate calls, also diminished program credibility and led to client frustration and mistrust. Several strategies (e.g., tracer being able to confirm a case’s date of birth, using Caller IDs) counteracted these tendencies and may have increased motivation to engage in contact tracing. While trust in health systems was often low, participants mentioned their pre-established relationships with known medical providers as reasons for engaging in contact tracing.

#### Emotional responses theme

Many participants described feeling shocked or anxious upon receiving a positive test result or exposure notification, and others anticipated being stigmatized by others in the community. These emotions could distract participants during the contact tracing call, but tracers who communicated clearly and empathically helped some remain calm. Others found the calls frustrating, particularly when they were numerous, duplicative, or timely. One participant “didn’t continue the call” because she received several calls from separate tracers due to an error in which she appeared in the database as multiple unique entries. Other emotions that affected participants during isolation/quarantine were loneliness and boredom. Coping strategies such as communicating electronically with family and friends and staying physically active mitigated such feelings and made isolation/quarantine more tolerable.

## Discussion

Contact tracing’s ability to reduce transmission of COVID-19 is limited due to many factors including short incubation periods and high transmission rates, yet contact tracing remains valuable by providing opportunities to establish and maintain trust in the health system through delivery of social support and education. This is one of the first studies to qualitatively examine the experiences of COVID-19 cases and contacts with contact tracing, and its findings may help to understand and address elements influencing participation in testing, answering phone calls, interviews, and isolation/quarantine. Low rates of contact tracing acceptance across jurisdictions in conjunction with our findings highlight the fact that many cases and contacts simply cannot or will not participate fully in this intervention, as the title of this article suggests. Below, we situate our findings within the contact tracing literature, and apply the Behavior Change Wheel to suggest solutions in the form of intervention functions targeting facilitators/barriers we identified in this study, to help increase client engagement with COVID-19 contact tracing (Table 4).

**Table 4:** Potential interventions and intervention functions within each COM-B Domain and Theme

COM-B Domains	Themes	Intervention Functions	Potential Intervention Activities	Behaviors
**Capability**	Symptom Severity	Enablement	Provide additional means of outreach and data collection (SMS, email, web application) for those with moderate-severe illness	A, P
			Monitor symptoms during Isolation and Quarantine and provide direct linkages to medical care	I/Q
	Essential Knowledge	Education	Increase community awareness of testing locations and COVID-19 symptoms	T
			Directly educate cases at time-of-testing that they will receive a contact tracing call	A
			Broadly educate the community that exposed contacts will receive a contact tracing call	A
			Educate clients (at testing sites and at the onset of contact tracing interviews) about the importance of contact tracing and how data will be protected	P
			Provide clear isolation and quarantine instructions	I/Q
**Opportunity**	Structural Context	Enablement	Provide transportation for testing	T
			Make testing free for those without health insurance, and advertise its availability	T
			Hire contact tracers fluent in common non-English languages and have interpreter services available	A, P
		Incentivization	Offer paid work leave	A, P, I/Q
		Training	Train contact tracers to screen for and identify resource needs and provide linkages to local resources	I/Q
	Interpersonal Ties	Modeling	Encourage and equip community members to promote engagement amongst peer groups	T, A, P, I/Q
			Encourage cases to inform their contacts that they will receive a contact tracing call, and provide suggestions about how to break the news	A, P
			Recruit community role models	T, A, P, I/Q
		Enablement	Establish a family point-of-contact to facilitate outreach when case is unavailable or unable to participate in the interview	A, P
			Help clients identify members of their social networks who may support them and give advice on how to break the news and seek help.	I/Q
**Motivation**	Symptom Severity	Persuasion	Emphasize potential harms of breaking isolation or quarantine among asymptomatic clients	I/Q
	Anticipated Outcomes	Incentivization	Screen for social and medical support needs, link clients to services, and advertise available resources.	T, A, P, I/Q
		Persuasion	Emphasize the benefits to family and/or community of participating fully in contact tracing	T, A, P, I/Q
	Trust in Authority	Persuasion	Establish trust in health systems and reduce fears regarding misuse of data via messaging campaigns that use peer- and provider-driven outreach; Caller ID; and representative, locally-based contact tracers	T, A, P, I/Q
	Emotional Responses	Enablement	Allow clients to select the frequency and mode of communication to avoid intrusion,	T, A, P, I/Q
			Equip tracers with a centralized and frequently updated database to reduce risk of frustrating cases and contacts with disorganized calls	P
			Advertise and provide access to hotlines, discussion forums, home-based activities	I/Q
		Persuasion	Use messages that emphasize the positive role one can play in protecting their community	P, I/Q
		Training	Equip contact tracers with skills to respond appropriately to client emotions during interview	P

**Abbreviations**

COM-B: Capability, Opportunity, Motivation, Behavior

T: Testing

A: Answering phone calls

P: Participating in interviews

I/Q: Isolation and quarantine

Our findings suggest that symptom severity and baseline knowledge influenced participants’ *Capability* to engage in contact tracing. Other studies similarly highlight the importance of community awareness in increasing adherence to health guidance both in the setting of COVID and elsewhere [13, 27]. However, physical symptoms may uniquely influence contact tracing for COVID-19, given the reliance on timeliness of tracing and propensity for symptoms to prevent engagement. This contrasts with contact tracing for sexually transmitted infections or TB in which incubation periods and end-goals of tracing differ, permitting more time to conduct contact tracing. Several potential intervention activities can address these elements. Alternative modes of data collection, such as digital communications or web-app surveys implemented in several U.S. states [28], could increase access to cases with moderate-to-severe symptoms. However, the use of such technology may be limited due to access to devices or technology literacy, and future research should further elucidate the potential impact of these methods. To increase engagement, programs could also improve health literacy by expanding community-wide education about COVID-19 and when and where to seek testing. When getting tested, individuals could be informed to expect and answer contact tracing calls should they test positive and be assured about data privacy and confidentiality concerns. Clear and standardized instructions on the duration and rules for isolation and quarantine might also improve adherence and reduce confusion, although continually evolving guidelines make this goal challenging. Given the difficulty that clients reported understanding and retaining this information, especially when receiving potentially upsetting news about a COVID-19 diagnosis or exposure, printed or electronic informational booklets could be provided at the time of testing or client interview.

Our data also suggest that environment and social ties strongly influenced *Opportunity* to engage in contact tracing. Consistent with previous literature [13, 14, 29], participants noted how access to medical care and support resources and social vulnerability influenced contact tracing behaviors. The data suggest that providing transportation to testing sites, offering home-testing, hiring multilingual contact tracers, offering paid work leave, supporting caregiving or urgent errand needs, and delivering care packages of food, masks, and cleaning supplies could all help promote contact tracing behaviors. Previous studies drawing on focus groups with COVID-19 contact tracers or with the general population support these strategies [23, 27]. However, cost may limit feasibility in some settings, and home-testing would also depend on clients reporting their results. Our qualitative analyses suggested that having cases notify contacts about what to expect from a contact tracing call and helping contacts identify peer resources to support isolation and quarantine can all be valuable. Communication between cases and contacts is often encouraged or relied upon in contact tracing for other communicable diseases [30] and may partially explain the previously observed correlations between success rates of contact outreach within case-contact clusters [22]. In contrast, social norms in some communities may reinforce a lack of adherence to COVID-19 health guidance [11, 29].

Last, our analysis suggested that symptom severity, anticipated outcomes, trust in health systems, and emotions could influence client *Motivation* to participate in contact tracing. Recent studies also emphasize that anticipated benefits of participation [14, 15, 29, 31] and trust in authority are important predictors of adherence to public health interventions [32, 33]. Transparent communications and strong patient-provider relationships can help build and maintain such trust [34–36] while misinformation [37] and privacy concerns [34] can undermine it. We also found that initiating tracing through known healthcare professionals or using Caller ID and addressing privacy concerns reinforced credibility and built trust, while redundant or uncoordinated efforts did the opposite. Potential interventions to increase trust in contact tracing indirectly supported by our analysis include hiring community members as contact tracers [38] and using peer- and provider-driven messaging campaigns to educate on the safety and purpose of contact tracing. We also observed that emotions affected motivation, including fear and anticipated stigma, as described with TB contact tracing [13]. Our analysis also suggests that equipping tracers with good communication skills is important. Training tracers to address shock or anxiety may help clients remain engaged when receiving test results or exposure notifications, and knowing how to elicit and address client needs is critical to success. Other interventions, stemming indirectly from the study findings, include connecting clients to mental health hotlines or online communication forums. Helping them remain active at home may decrease loneliness and boredom associated with isolation and quarantine. Furthermore, improving the coordination of outreach efforts and allowing clients choices in the method and frequency of outreach may enhance engagement.

**Fig. 1:**
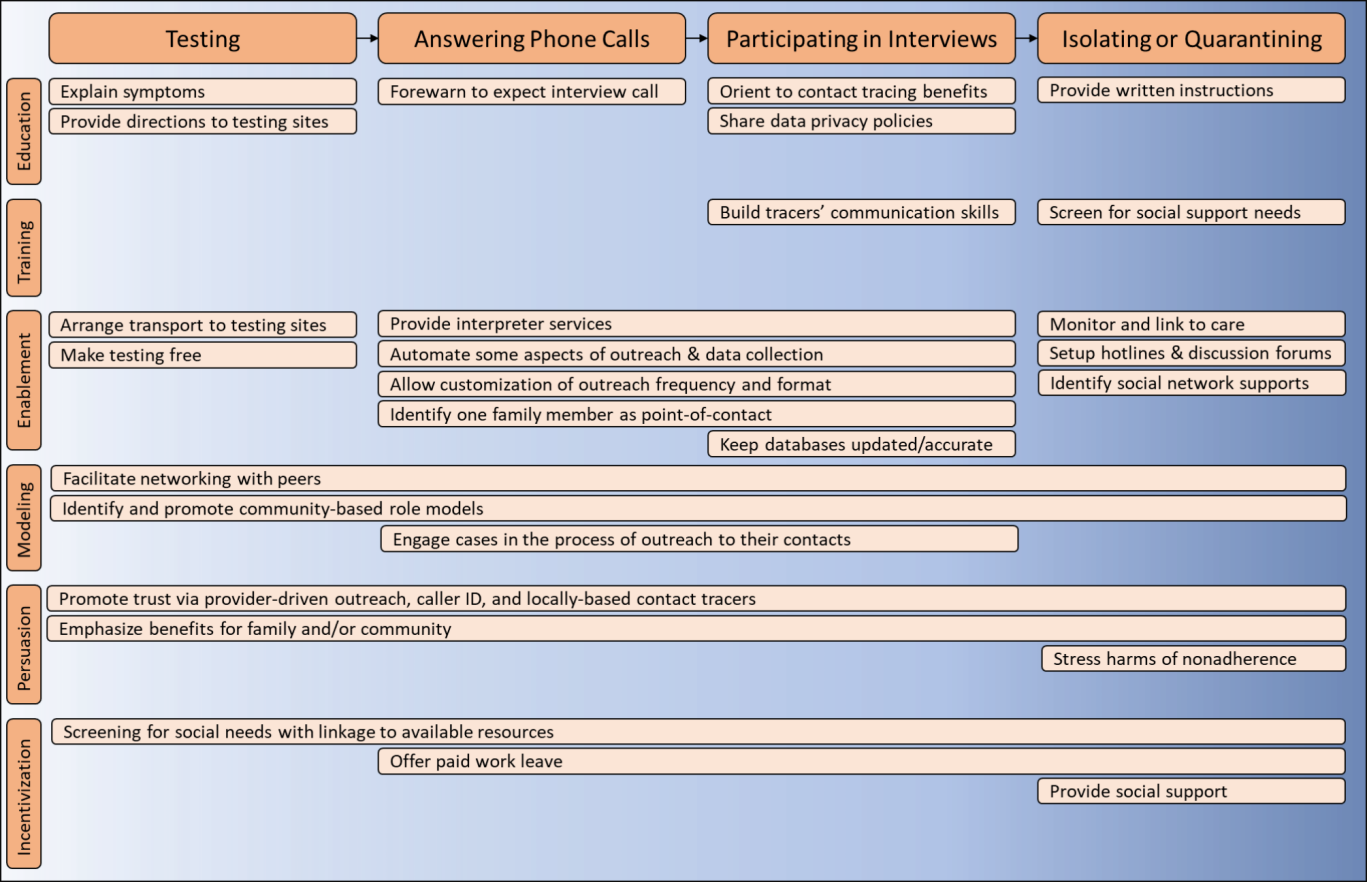
Suggested interventions mapped according to sequential behaviors they might affect (top) and Behavior Change Wheel function (left)

Using COM-B in this study enhanced the utility of our findings by connecting the identified barriers and facilitators and possible interventions to the Behavior Change Wheel [17]. Some of the potential mechanisms for influencing change are shown for the interventions proposed in Table 4, including (1) Enablement (i.e., increasing means of engagement or reducing barriers), (2) Education (i.e., increasing knowledge), (3) Persuasion, (i.e., using communication to create positive or negative feelings), (4) Modeling, (i.e., providing an example of desired behavior), (5) Training, (i.e., imparting new skills), and (6) Incentivization, (i.e., establishing an expectation of reward). Figure 1 displays the suggested interventions mapped according to their function and the contact tracing behaviors they might affect, based upon our analysis. Of note, interventions affecting multiple behaviors or affecting earlier steps in the contact tracing process may be more beneficial than interventions affecting only single or downstream behaviors. Future implementation and evaluation of these interventions should consider feasibility and acceptability of each function based on local context and resources.

### Limitations and strengths

There are several limitations to this study. First, poor recall of experiences over time may have introduced some inaccuracies in the data, though we sought to minimize this by interviewing clients soon after their original contact tracing call. Second, social desirability or response biases may have influenced participants to present themselves in positive terms, although interviewers were trained to be non-judgmental towards and supportive of participants to minimize this possibility. Third, although we achieved data saturation, we recruited participants from a single contact tracing program, and no data were available from clients who were not reached by or declined to participate with the program. This limits the transferability of our findings to different settings or populations with lower baseline health engagement (i.e., those who never answered contact tracer calls). Fourth, we acknowledge that the relationships between themes are not fully elucidated by our analysis, reflecting a potential limitation of COM-B and the Behavior Change Wheel to synthesize the insights gained from this study.

This study is strengthened by its use of qualitative data collected at the onset of the COVID-19 pandemic. This time period was critical in shaping the course of the pandemic and learning from the experiences and decisions within this timeframe is important for future improvement. While many quantitative contact tracing studies have been published, few provide the reasons why clients fail to engage. Eliciting participant experiences in their own words adds strength to findings reported elsewhere and also yielded new insights into the complexities of increasing contact tracing uptake, such as the role of social network communication in shaping contact tracing behaviors. Another strength is the inclusion of both cases and contacts, as well as both English and Spanish speakers. A final strength is the use of the COM-B model to frame the analysis and findings. This approach allowed us to link our identified themes with relevant COM-B domains and potential intervention activities, many of which are transferable to other similar settings.

## Conclusion

This study is among the first to explore, from the perspective of COVID-19 cases and contacts, how their environments, experiences, and perceptions may shape contact tracing behaviors. Within the COM-B framework, *Capability* was shaped by symptom severity and COVID-19-relevant knowledge, *Opportunity* was shaped by structural, environmental, and social factors, and *Motivation* was shaped by symptoms, anticipated consequences of engagement, trust, and emotional responses. Tracing strategies may benefit from accounting for and addressing the many environmental- and client-level elements identified herein, and clients’ symptoms and peer interactions may be more influential in the setting of COVID-19 contact tracing compared to other disease contexts.

## Electronic supplementary material

Below is the link to the electronic supplementary material.

Supplementary Material 1

## Data Availability

Data may be made available upon reasonable request to the New Haven Health Department.
