# Supplementary material for: “I can’t do it”: A qualitative study exploring case and contact experiences with COVID-19 contact tracing
Source: BMC Public Health. 2022 Oct 25;22:1963. doi: 10.1186/s12889-022-14265-8 (PMC9595089; doi:10.1186/s12889-022-14265-8)
Supplement: Supplementary file 1 — Supplementary Material 1 [file 12889_2022_14265_MOESM1_ESM.docx]

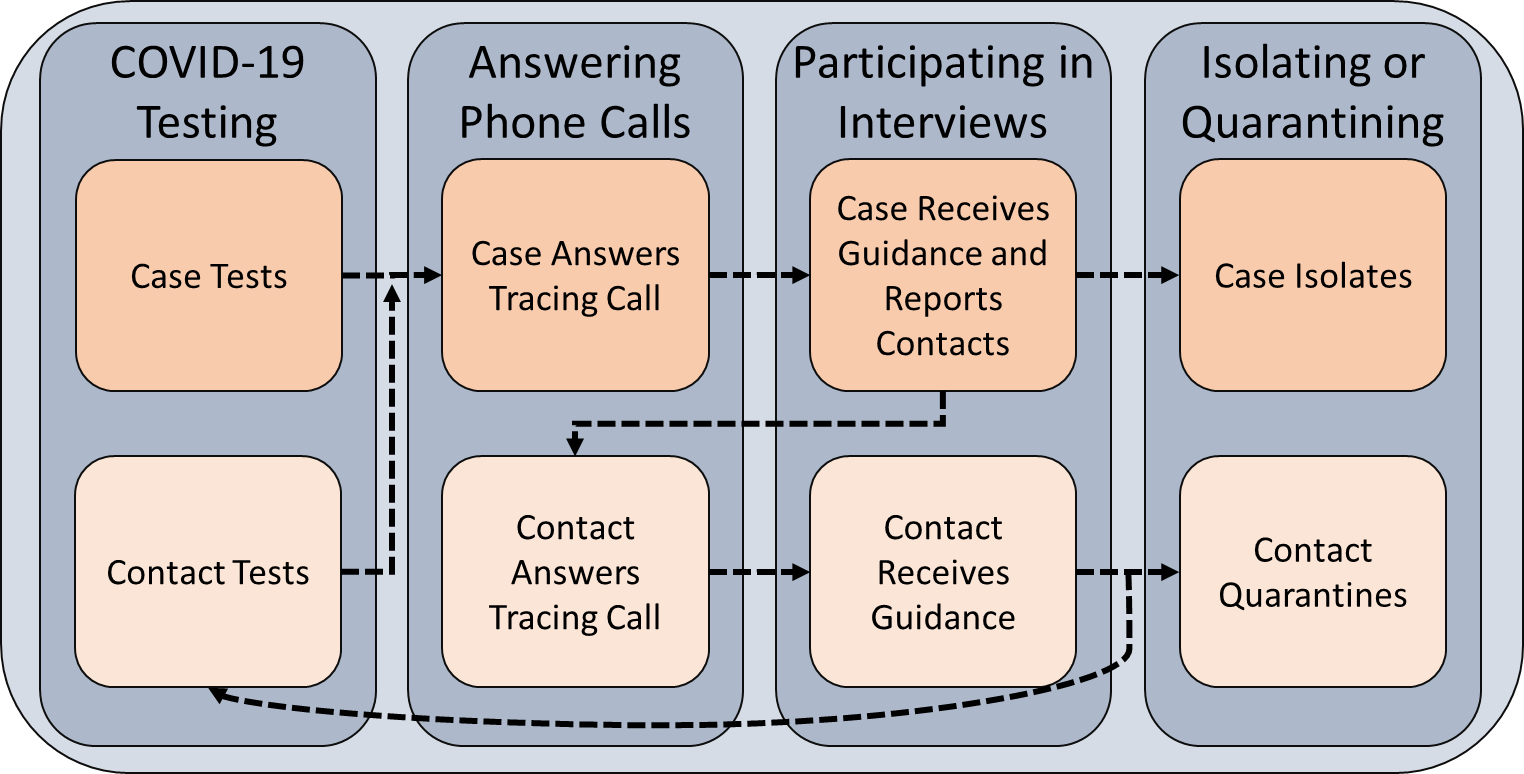


**Supplementary Figure 1**: Conceptual model of the four contact tracing behaviors, differentiated for cases (dark orange) and contacts (light orange). Dashed arrows are used to indicate that not all clients proceed between each step.
